# Effects of subthalamic deep brain stimulation on striatal metabolic connectivity in a rat hemiparkinsonian model

**DOI:** 10.1242/dmm.039065

**Published:** 2019-05-24

**Authors:** Nadine Apetz, Elena Kordys, Mascha Simon, Britta Mang, Markus Aswendt, Dirk Wiedermann, Bernd Neumaier, Alexander Drzezga, Lars Timmermann, Heike Endepols

**Affiliations:** 1 University of Cologne, Faculty of Medicine and University Hospital Cologne, Institute of Radiochemistry and Experimental Molecular Imaging, Kerpener Str. 62, 50937 Köln, Germany; 2 Max Planck Institute for Metabolism Research, Department of In-vivo NMR, Gleueler Str. 50, 50931 Köln, Germany; 3 Forschungszentrum Jülich GmbH, Institute of Neuroscience and Medicine, Nuclear Chemistry (INM-5), Wilhelm-Johnen-Straβe, 52428 Jülich, Germany; 4 University of Cologne, Faculty of Medicine and University Hospital Cologne, Department of Nuclear Medicine, Kerpener Str. 62, 50937 Köln, Germany; 5 University of Cologne, Faculty of Medicine and University Hospital Cologne, Department of Neurology, Kerpener Str. 62, 50937 Köln, Germany

**Keywords:** Hemiparkinsonian rat model, Positron emission tomography, Dopamine, Connectivity, Deep brain stimulation

## Abstract

Deep brain stimulation (DBS) in the subthalamic nucleus (STN) has been successfully used for the treatment of advanced Parkinson's disease, although the underlying mechanisms are complex and not well understood. There are conflicting results about the effects of STN-DBS on neuronal activity of the striatum, and its impact on functional striatal connectivity is entirely unknown. We therefore investigated how STN-DBS changes cerebral metabolic activity in general and striatal connectivity in particular. We used ipsilesional STN stimulation in a hemiparkinsonian rat model in combination with [^18^F]FDOPA-PET, [^18^F]FDG-PET and metabolic connectivity analysis. STN-DBS reversed ipsilesional hypometabolism and contralesional hypermetabolism in hemiparkinsonian rats by increasing metabolic activity in the ipsilesional ventrolateral striatum and by decreasing it in the contralesional hippocampus and brainstem. Other STN-DBS effects were subject to the magnitude of dopaminergic lesion severity measured with [^18^F]FDOPA-PET, e.g. activation of the infralimbic cortex was negatively correlated to lesion severity. Connectivity analysis revealed that, in healthy control animals, left and right striatum formed a bilateral functional unit connected by shared cortical afferents, which was less pronounced in hemiparkinsonian rats. The healthy striatum was metabolically connected to the ipsilesional substantia nigra in hemiparkinsonian rats only (OFF condition). STN-DBS (ON condition) established a new functional striatal network, in which interhemispheric striatal connectivity was strengthened, and both the dopamine-depleted and the healthy striatum were functionally connected to the healthy substantia nigra. We conclude that both unilateral dopamine depletion and STN-DBS affect the whole brain and alter complex interhemispheric networks.

## Abstract (precis)

**Summary:** Deep brain stimulation in the subthalamic nucleus in rats with a unilateral dopaminergic lesion established a new functional interhemispheric striatal network.

## INTRODUCTION

Deep brain stimulation (DBS) in the subthalamic nucleus (STN) has been successfully used for the treatment of advanced Parkinson's disease (PD) since the 1990s (Benabid et al., 1994; Krack et al., 1997; Limousin et al., 1995). The underlying mechanisms are complex and not well understood. At the stimulation site itself, DBS effects go beyond the excitation of fibers and inhibition of cell bodies, by inducing novel dynamic states in the stimulated neuronal elements (Hamani et al., 2017). Functional connectivity analyses in DBS-treated PD patients have recently shown that STN-DBS affects the whole basal ganglia-thalamus-cortical loop, disrupts pathological oscillations and strengthens connections with the cerebellum (Ashkan et al., 2017; Mueller et al., 2018). However, classical functional magnetic resonance imaging (fMRI) studies of DBS effects are limited, because there is a risk of overheating or displacement of the electrodes. Furthermore, the stimulator has to be kept at a distance from the magnet, thus fMRI can only be done in the few days between electrode placement and stimulator implantation (Albaugh and Shih, 2014; Jech et al., 2001). To overcome these limitations, functional connectivity analyses based on positron emission tomography (PET) studies with the tracer 2-deoxy-2-[^18^F]fluoroglucose ([^18^F]FDG) have been developed (Yakushev et al., 2017). The concept of this so-called ‘metabolic connectivity analysis’ is based on the correlation of [^18^F]FDG uptake in a seed region with all other voxels of the brain across subjects, and has been demonstrated in humans (Sala et al., 2017; Verger et al., 2018) but recently also in rats (Liang et al., 2018; Rohleder et al., 2016). Other than conventional MRI-based functional connectivity analysis, FDG-PET-based metabolic connectivity analysis does not interfere with DBS. It has been shown using this method that internal globus pallidus (GPi)-DBS reduced the activity of ipsilateral PD-related metabolic covariance patterns, which correlated with improvement of motor symptoms (Fukuda et al., 2001).

Functional connectivity of the striatum is severely altered in PD. In general, connectivity with subcortical areas (thalamus, midbrain, pons) is diminished, whereas both decreases and increases for different cortico-striatal connections have been reported (Hacker et al., 2012; Hou et al., 2016; Manza et al., 2016). Conflicting results about the effects of STN-DBS emerged from imaging studies: some authors reported STN-DBS-related activations of the striatum (Geday et al., 2009; Hilker et al., 2004; Knight et al., 2015; Stefurak et al., 2003), whereas others found deactivations (Cao et al., 2017; Le Jeune et al., 2010). The impact of STN-DBS on striatal connectivity is entirely unknown. Furthermore, metabolic connectivity in animal models of PD and changes resulting from DBS have not yet been examined.

So far, STN-DBS in rat models has been employed in combination with extensive behavioral testing (He et al., 2014; Li et al., 2010; Lindemann et al., 2012; Vlamings et al., 2007), electrophysiological recordings (Anderson et al., 2015; Chuang et al., 2018; Gubellini et al., 2006; Hartung et al., 2011; Li et al., 2010; McConnell et al., 2012; Polar et al., 2018; Shi et al., 2006; Sutton et al., 2015; Temel et al., 2007), microdialysis (Lacombe et al., 2007), analysis of neuronal activity markers or neurotrophic factors (Chuang et al., 2018; Lacombe et al., 2009; Spieles-Engemann et al., 2011; Tan et al., 2011) and imaging (Klein et al., 2011; Lai et al., 2014). This work is part of a larger study about the effects of STN-DBS in a unilateral 6-hydroxydopamine (6-OHDA) rat model of Parkinson's disease. Dopamine depletion measured with 6-[^18^F]fluoro-l-dopa ([^18^F]FDOPA)-PET, brain metabolism in the OFF state measured with [^18^F]FDG-PET and gait analysis (OFF) were described in an earlier publication (Kordys et al., 2017). We have shown that unilateral 6-OHDA lesions lead to ipsilesional hypometabolism and contralesional hypermetabolism, and that both phenomena are associated with motor impairments as well as compensation (Kordys et al., 2017). Here, we examined in the same animals how STN-DBS (ON state) changes focal metabolic activity in relation to dopamine depletion severity, and how it affects striatal connectivity.

## RESULTS

### DBS effects on cerebral [^18^F]FDG uptake

**Fig. 1.**
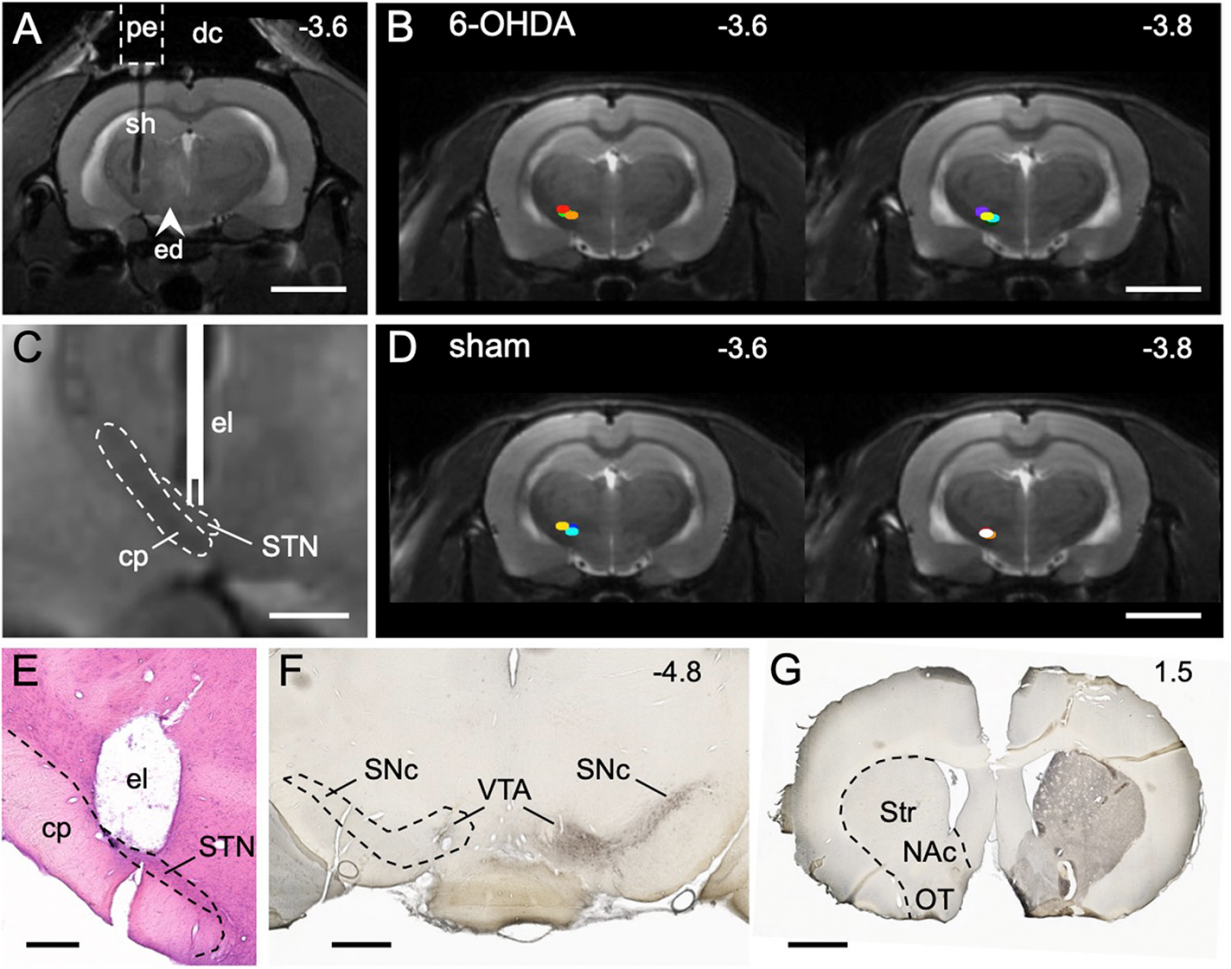
**STN stimulation site and dopaminergic lesion.** (A) MRI (T2) 24 h after implantation of the guide cannula and 6-OHDA injection. The pedestal (pe) of the guide cannula is attached to the skull with dental cement (dc). The shaft (sh) targets the STN. A faint edema (ed) is visible from 6-OHDA injection into the medial forebrain bundle. (B) Stimulation sites (colored dots) of 6-OHDA animals (*n*=7). (C) MRI detail from the stimulation site with sketched electrode (el). The cerebral peduncle (cp), which contains descending motor fibers, lies next to the STN and should not be stimulated. (D) Stimulation sites (colored dots) of sham animals (*n*=6). (E) Histological section showing the stimulation site after removal of the electrode. (F) TH immunostaining (transverse section level) demonstrating the loss of dopaminergic cell bodies in the left substantia nigra pars compacta (SNc) and ventral tegmental area (VTA) in a rat injected with 6-OHDA. (G) TH immunostaining (transverse section level) showing the loss of dopaminergic axon terminals in the left striatum (Str), nucleus accumbens (NAc) and olfactory tubercle (OT). Numbers are rostrocaudal coordinates (mm) relative to Bregma. Dashed outlines indicate borders of brain regions. Scale bars: 5 mm in A,B,D; 1 mm in C,F,G; 500 µm in E.

**Fig. 2.**
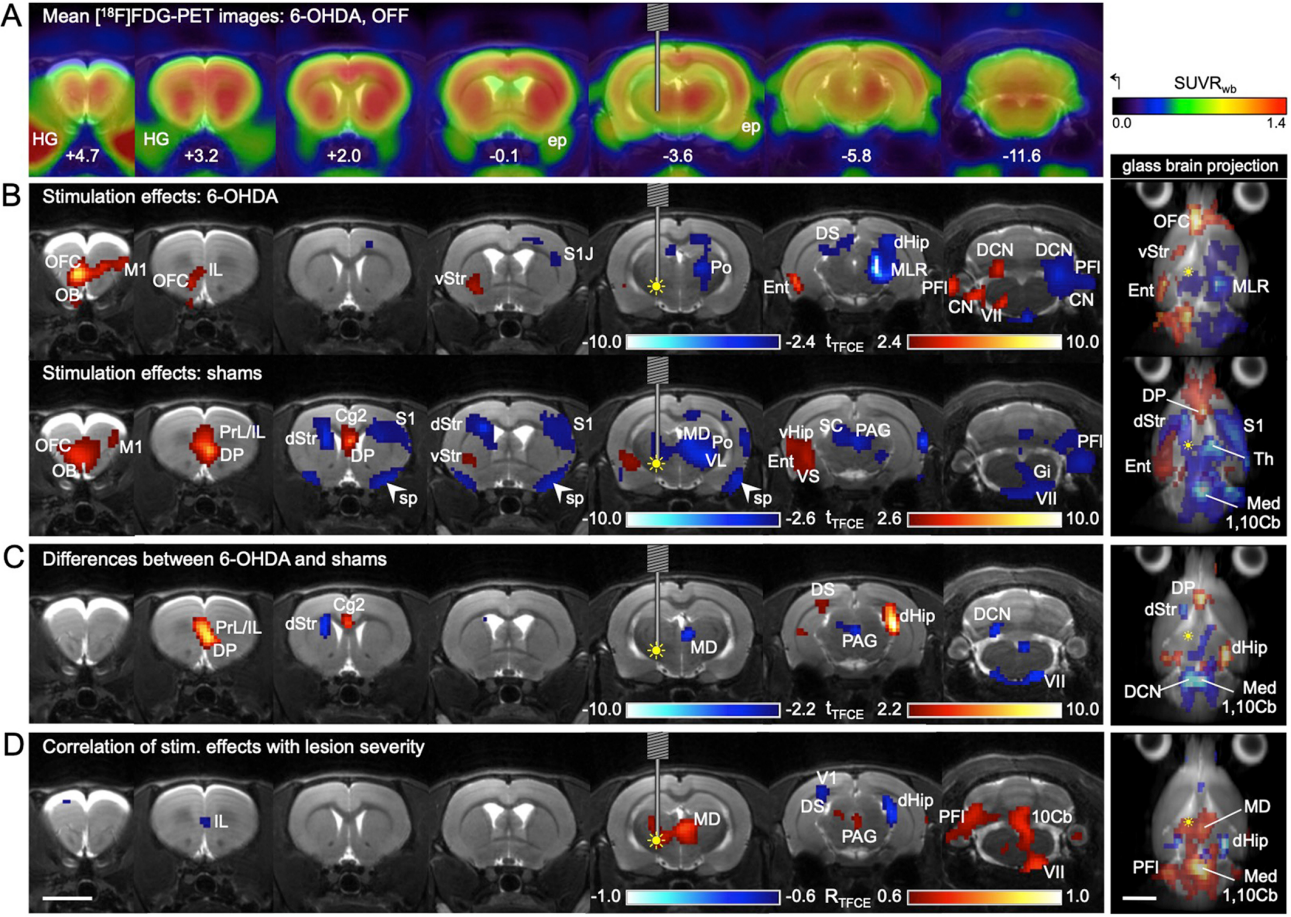
**Effects of STN-DBS on [^18^F]FDG uptake.** (A-D) Each column shows a transverse section level indicated by the rostrocaudal coordinates relative to Bregma in panel A. The different statistical analyses are presented in rows with a glass brain projection at the end. In the glass brain projection, all layers of significant voxels are visible, providing an overview of the whole brain. (A) Mean [^18^F]FDG images (*n*=6) of 6-OHDA animals with DBS-OFF were projected onto an MRI template. (B) The effects of DBS-ON on [^18^F]FDG uptake were analyzed separately in 6-OHDA (*n*=7, first row) and sham animals (*n*=6, second row) using a paired *t*-test. Red voxels: [^18^F]FDG uptake ON>OFF. Blue voxels: [^18^F]FDG uptake OFF>ON. (C) Because patterns of [^18^F]FDG uptake changes were not identical in the two groups, difference images ([^18^F]FDG uptake ON minus OFF=DBS effects) were compared between 6-OHDA rats and shams using a *t*-test. Red voxels, DBS effects sham>DBS effects 6-OHDA. Blue voxels, DBS effects 6-OHDA>DBS effects sham. (D) To assess whether DBS effects are related to dopamine depletion severity, a Pearson correlation analysis between DBS effects and reduction of striatal [^18^F]FDOPA uptake was performed using 6-OHDA rats and shams pooled (*n*=13). Red voxels, DBS effects were positively correlated with lesion severity. Blue voxels, DBS effects were negatively correlated with lesion severity. Yellow filled circle indicates inserted electrode and ongoing stimulation (DBS ON). 1,10Cb, first and tenth cerebellar lobule; Cg2, cingulate cortex 2; CN, cochlear nucleus; DCN, deep cerebellar nuclei; dHip, dorsal hippocampus; DP, dorsal peduncular cortex; DS, dorsal subiculum; dStr, dorsal part of dorsal striatum; Ent, entorhinal cortex; ep, external pterygoid muscle; Gi, gigantocellular reticular nucleus; HG, Harderian gland; IL, infralimbic cortex; M1, primary motor cortex; MD, mediodorsal thalamus; Med, medulla oblongata; MLR, midbrain locomotor region; OB, olfactory bulb; OFC, orbitofrontal cortex; PAG, periaqueductal gray; PFl, paraflocculus; Po, posterior thalamic nucleus; PrL, prelimbic cortex; S1, primary sensory cortex; S1J, jaw area of S1; SC, superior colliculus; sp, spill-over from ep; Th, thalamus; V1, primary visual cortex; VII, facial nucleus; vHip, ventral hippocampus; VL, ventrolateral thalamic nucleus; VS, ventral subiculum; vStr, ventral part of dorsal striatum. Scale bars: 5 mm.

Correct placement of the electrode as well as effectiveness of the 6-OHDA dopaminergic lesion was confirmed using histology and immunohistochemistry (Fig. 1). In both 6-OHDA and sham animals, STN-DBS generally increased [^18^F]FDG uptake in the frontal parts of the brain and in the ipsilesional hemisphere, whereas it decreased [^18^F]FDG uptake in the contralesional hemisphere (Fig. 2B). An increase of [^18^F]FDG uptake occurred bilaterally in the olfactory bulb and the orbitofrontal cortex, the contralateral primary motor cortex (M1), the ventrolateral part of the ipsilateral striatum and the ipsilateral entorhinal cortex. [^18^F]FDG uptake decreased in the contralateral sensory cortex, contralateral posterior thalamus and contralateral paraflocculus of the cerebellum (Fig. 2B).

Apart from this common pattern, we also found differences between groups, which are shown in Table 1 and Fig. 2C. The glass brain projection (Fig. 2C) demonstrates that group differences are distributed symmetrically across the brain.

**Table 1.**
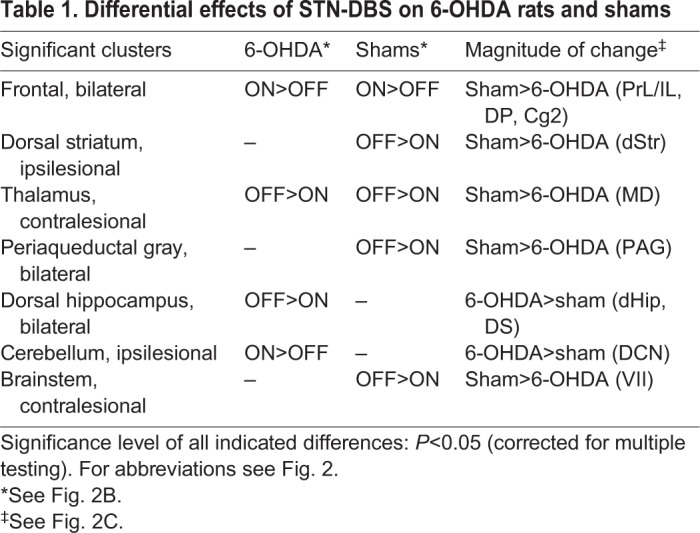
**Differential effects of STN-DBS on 6-OHDA rats and shams**

### Correlation of [^18^F]FDG uptake changes with lesion severity

Correlation analysis showed that some of the DBS effects on metabolism were related to dopamine depletion severity (Fig. 2D). Considering the direction of changes described above, four types of correlation can be defined, which are shown in Table 2.

**Table 2.**
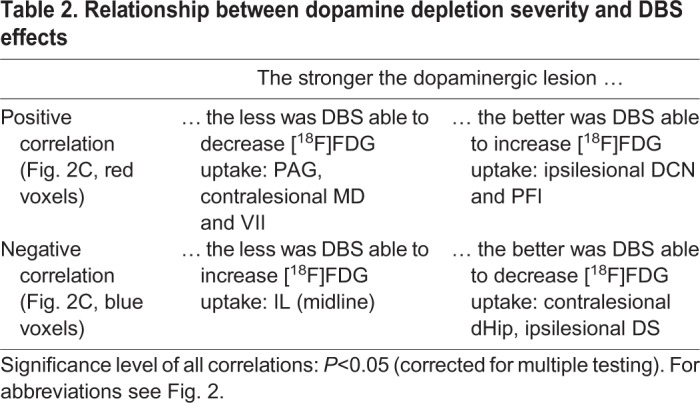
**Relationship between dopamine depletion severity and DBS effects**

The type ‘the stronger the dopaminergic lesion, the less was DBS able to decrease [^18^F]FDG uptake’ was predominant and occurred in the mediodorsal thalamus (contralesional), periaqueductal gray, cerebellum and hindbrain.

### Metabolic connectivity of the striatum

#### Positive correlations

**Fig. 3.**
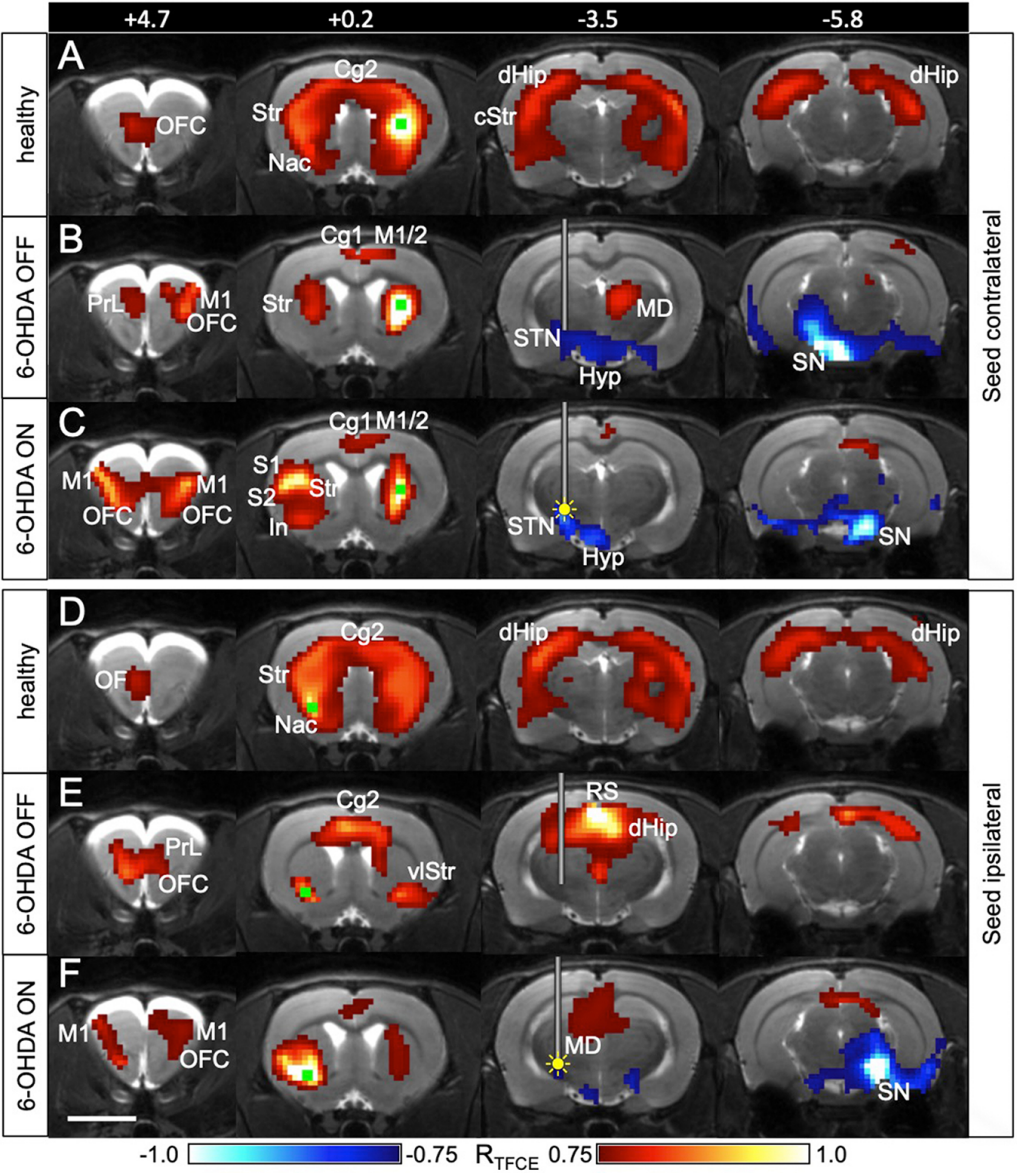
**Striatal connectivity is changed by STN-DBS.** (A-C) Connections of the healthy striatum are changed by STN-DBS. The seed (green square) was placed contralateral to the 6-OHDA injection in 6-OHDA animals (*n*=7) and in the right striatum in healthy controls (*n*=19). A Pearson correlation analysis was performed between the seed region and all other voxels of the brain. Yellow filled circle indicates inserted electrode and ongoing stimulation (DBS ON). (D-F) Connections of the dopamine-depleted striatum are changed by STN-DBS. The seed (green square) was placed ipsilateral to the 6-OHDA injection in 6-OHDA animals and in the left ventrolateral striatum in healthy controls. It was located exactly at the spot where STN-DBS increased [^18^F]FDG uptake. Red voxels, positively correlated with seed region with respect to [^18^F]FDG uptake. Blue voxels, negatively correlated with seed region. Numbers represent rostrocaudal coordinates (mm) relative to Bregma. The implanted guide cannula is shown in B,C,E,F. In A and D, no cannula was implanted. Cg1, cingulate cortex 1; Cg2, cingulate cortex 2; cStr, caudal striatum; dHip, dorsal hippocampus; Hyp, hypothalamus; In, insula; M1, primary motor cortex; M2, secondary motor cortex; MD, mediodorsal thalamus; Nac, nucleus accumbens; OF, orbitofrontal cortex; PrL, prelimbic cortex; RS, retrosplenial cortex; S1, primary sensory cortex; S2, secondary sensory cortex; SN, substantia nigra; STN, subthalamic nucleus; Str, striatum; vlStr, ventrolateral striatum. Scale bar: 5 mm.

Positive correlations between the seed region and other brain areas indicate that when the seed region shows high [^18^F]FDG uptake, the positively connected areas also display high [^18^F]FDG uptake, and vice versa. In healthy control animals, [^18^F]FDG uptake of the whole basal ganglia (ipsi- and contralateral) was positively correlated to [^18^F]FDG uptake of the seed region in the middle of the right striatum (Fig. 3A,D). When the seed was in the left ventrolateral striatum, the connectivity pattern was almost identical (Fig. 3D), suggesting that the basal ganglia form a bilateral functional unit. The dorsal hippocampus (ipsi- and contralateral) was metabolically connected to both striatal seed regions as well.

In 6-OHDA animals, the striatal cluster was smaller and the connection between the healthy striatum and its dopamine-depleted counterpart was weaker (Fig. 3B,E) during the OFF condition. With DBS ON, the contralateral connection was strengthened and appeared to be more widespread (Fig. 3C,F). When the seed was placed at the spot where DBS increased [^18^F]FDG uptake, the ipsilateral and contralateral clusters increased considerably in size. This indicates that DBS improves inter- and intrahemispheric striatal connectivity.

#### Negative correlations

Negative correlations between the seed region and other brain areas indicate that when the seed region has a high [^18^F]FDG uptake, the negatively connected areas have a low [^18^F]FDG uptake, and vice versa. In 6-OHDA animals, a strong negative correlation during OFF was present between the seed in the healthy striatum and the contralateral dopamine-depleted substantia nigra (SN) (Fig. 3B), which was completely absent in healthy control animals. In contrast, the dopamine-depleted ventrolateral striatum was not connected to SN on either hemisphere. When DBS was switched ON, both striatal seeds, the contralesional (healthy) and the ipsilesional (dopamine-depleted) one, showed a negative connection to the SN region on the healthy side (Fig. 3C,F).

## DISCUSSION

### DBS effects on metabolic activity

We used [^18^F]FDG-PET to measure DBS-induced changes in focal brain activity in a unilateral rat model of PD. Cerebral [^18^F]FDG uptake is a surrogate marker for glucose consumption and reflects synaptic activity (Dienel et al., 2018; Raichle and Mintun, 2006; Schwartz et al., 1979). In a previous study (Kordys et al., 2017) we have described that unilateral 6-OHDA injection into the medial forebrain bundle causes ipsilesional hypometabolism in the striatum and thalamus, and contralesional hypermetabolism in the striatum and midbrain locomotor region. Here, we show that STN-DBS reversed these changes by increasing metabolic activity in many ipsilesional brain regions and by decreasing it on the contralesional side of 6-OHDA animals. Most importantly, DBS increased metabolic activity in the ipsilesional ventrolateral striatum. This appears to be a stable effect as it occurred in sham animals as well, and was also described in another rat PET study (Klein et al., 2011). Furthermore, STN-DBS strongly decreased metabolic activity in the contralesional thalamus and midbrain of 6-OHDA animals. These results indicate that STN-DBS counteracts the metabolic imbalance between brain hemispheres caused by unilateral 6-OHDA injection.

Our PET data also showed that some STN-DBS effects were subject to the magnitude of dopaminergic lesion severity. The most prominent result was that the widespread metabolic deactivations seen in sham animals, particularly those on the ipsilesional side, were far less pronounced in 6-OHDA animals. Although similar data from human PD patients are hardly available as DBS is preferentially used in the late stages of PD with strong dopamine depletion, differential effects of DBS depending on disease severity have also been discussed for PD patients (Stefani et al., 2018).

### Positive functional connectivity

**Fig. 4.**
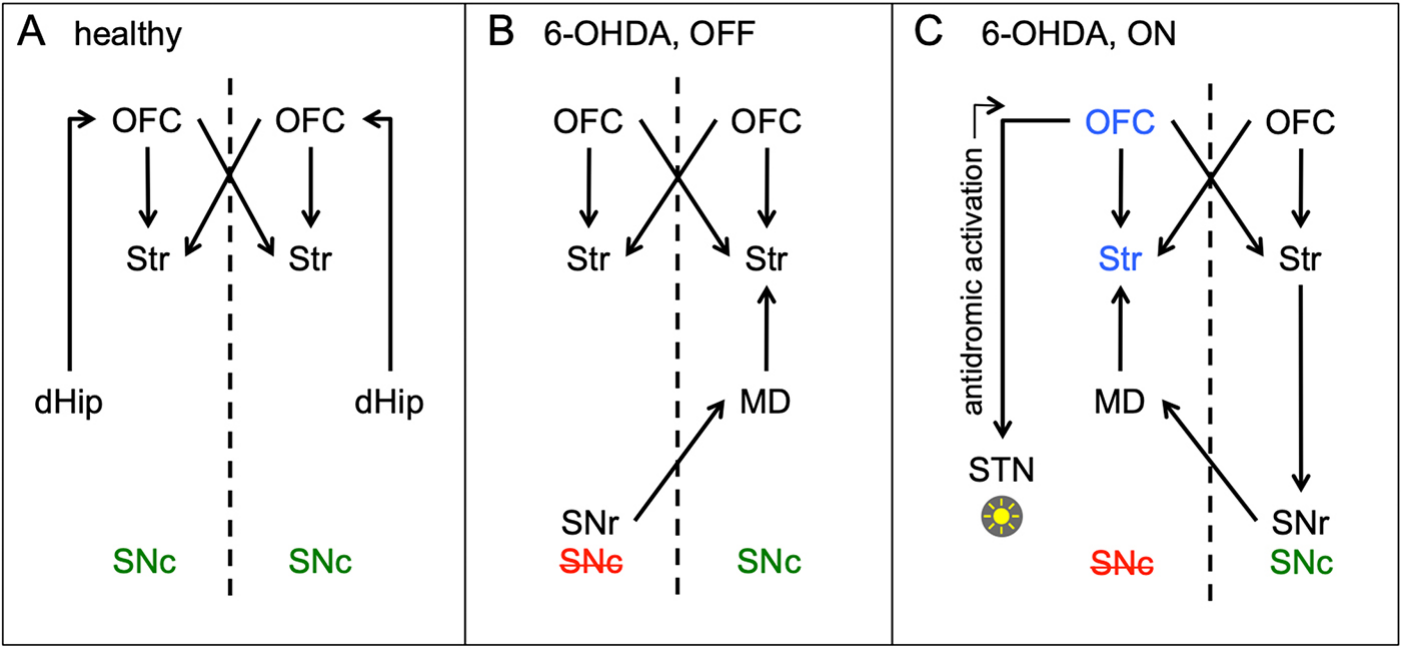
**Hypothetical connections of the striatal network.** (A-C) The striatal network is shown in healthy animals (A), 6-OHDA animals in the DBS OFF state (B) and in the DBS ON state (C). Areas that are significantly activated by STN-DBS are indicated in blue. Color of SNc indicates healthy (green) and lesioned side (red).

Functional connectivity assessment with [^18^F]FDG-PET is based on one cumulative metabolic value measured per voxel in a number of animals, rather than on the time course of a physiological signal (e.g. BOLD fluctuations) per voxel in one animal. Across a group of rats, we analyzed the correlation between the metabolic value of a small seed region and all other voxels in the brain. Unlike in fMRI-based functional connectivity analysis, the exact timing of input activity plays no role for the correlation of [^18^F]FDG-PET signals. Therefore, connections with complex time-lag structures, which may be missed by fMRI data (Meszlényi et al., 2017), will be captured by PET. Direct anatomical connections are often present between functionally connected brain areas, but are not essential. Rather, functionally linked areas may be serially connected through a third region or share common inputs (Adachi et al., 2012). The latter may be the case for the bilateral basal ganglia cluster we observed in healthy rats (Fig. 4A). Neither striatum nor nucleus accumbens has direct interhemispheric connections, but share bilateral inputs from the cortex (Wilson, 1987), which becomes evident as a cingular and orbitofrontal ‘bridge’ between left and right striatum. Furthermore, we found that the dorsal hippocampus was bilaterally connected to the striatum in healthy rats, also via the orbitofrontal cortex (Brown et al., 2012). In 6-OHDA animals, striatal interhemispheric connections were less extensive, which can be interpreted as weakening of striatal input activity. It has been shown that the lack of dopamine leads to reduced firing of cortical pyramidal tract neurons, which send collaterals into the striatum (Pasquereau and Turner, 2011; Shepherd, 2013). These connections may have been strengthened by STN-DBS, which has previously also been shown with thalamic DBS (Lin et al., 2015).

### Negative functional connectivity

Although negative functional connectivity values are frequently observed in fMRI network analyses, their biological relevance is unclear. Negative correlations between brain areas were long thought to be artifacts caused by methodological peculiarities of fMRI analysis or by brain hemodynamics (Chen et al., 2011; Parente et al., 2018). These explanations do not account for [^18^F]FDG-PET seed-based analyses, as those are not influenced by short-term hemodynamics or time series artifacts. Furthermore, there is recent evidence that negative correlations are real biological phenomena, which increase during brain maturation (de Lacy et al., 2018; Gee et al., 2013) and that they reflect regulatory interactions between brain regions, such as reciprocal modulations, suppression, inhibition and neurofeedback (Gopinath et al., 2015). In 6-OHDA animals during the DBS OFF state, we observed a negative correlation between the seed in the contralesional striatum and the ipsilesional SN region. This indicates that the higher metabolism was in the contralesional striatum, the lower metabolism was in the ipsilesional SN region and vice versa. A similar shift towards negative correlations between striatal nuclei and the ‘extended brainstem’ (which includes the midbrain) has been described in a resting state fMRI study with PD patients (Hacker et al., 2012). One explanation for our rats could be that a low SN metabolic signal simply reflects dopaminergic cell death in the substantia nigra pars compacta (SNc) and hence dopamine depletion severity, which causes contralesional striatal hypermetabolism (Kordys et al., 2017). This scenario would not necessarily require a neuronal network connection between the ipsilesional midbrain and the contralesional striatum. However, we did not find hypometabolism in the ipsilesional midbrain in 6-OHDA animals (Kordys et al., 2017) and STN-DBS, which abolished the abovementioned correlation, did not increase metabolic activity in the SN region. This rules out a strong influence of non-synaptic factors (i.e. dopaminergic cell death and/or DBS) on the metabolic activity of the ipsilesional midbrain. The negative correlation may therefore be driven by synaptic activity within a bilateral basal ganglia network. In this case, a neuronal network connection between the dopamine-depleted midbrain and the contralesional striatum must exist. According to our data, the mediodorsal thalamus (MD) may be part of this network. We found that the contralesional MD was positively connected with the contralesional striatum during the DBS OFF state. The MD receives intensive projections from the contralateral substantia nigra pars reticulata (SNr) (Gerfen et al., 1982) and is anatomically connected with the striatum via the globus pallidus (Mitchell and Chakraborty, 2013). Our data therefore suggest that there is a strong functional connection between the SNr of the lesioned side and the striatum of the healthy hemisphere via the contralesional MD in hemiparkinsonian rats (Fig. 4B). In the DBS ON state, the negative correlation between the contralesional striatal seed and the ipsilesional SN region disappeared. Likewise, the contralesional MD was no longer part of the network. Instead, a negative correlation between the contralesional striatum and the contralesional (healthy) SN emerged.

To explain how STN-DBS is able to induce this shift of functional connections we placed a second seed in the ipsilesional ventrolateral striatum, at exactly the same spot where DBS increased metabolic activity. In the DBS ON state, we observed a negative correlation with the contralesional SN, which was not present in the OFF state. The correlation pattern looked almost exactly like the pattern seen with the contralesional seed. As explained before, crossed connections may have been relayed through the MD, which was also part of the ipsilesional ventrolateral striatal network. However, if we assume that the flow of information has to start in the stimulated hemisphere, the contralateral SNr could not have been directly recruited via the MD, which receives afferents from the contralateral SNr, but does not send efferents back (Mitchell and Chakraborty, 2013). Rather, a possible way for DBS-induced neuronal activation actually passing to the contralateral hemisphere was via the orbitofrontal cortex, which projects to the STN and may have been activated through antidromic stimulation (Li et al., 2014) (Fig. 4C). In both 6-OHDA and sham animals we found a bilateral activation of the orbitofrontal cortex during DBS, extending into the contralateral rostral M1. Both areas sent excitatory projections to the ipsilateral striatum (Deng et al., 2015; Mailly et al., 2013) and participated in ipsi- and contralesional striatal networks in the DBS ON state. In turn, contralesional striatal efferents may have inhibited the high spontaneous activity of the contralesional SNr (Chevalier and Deniau, 1990). Information flow via the ipsilesional MD back to the ipsilesional striatum may have closed the loop, which can explain the clusters of negative correlation contralateral to the stimulation with both ipsi- and contralesional seeds.

We conclude that in hemiparkinsonian rats the healthy striatum was functionally connected to the ipsilesional SN. STN-DBS established a new functional interhemispheric striatal network, in which both the dopamine-depleted and the healthy striatum were functionally connected to the healthy SN. This demonstrates that both unilateral dopamine depletion and STN-DBS affect the whole brain and alter complex interhemispheric networks. Whether the DBS-induced nigral connectivity shift can be interpreted as an improvement is unclear, as naive rats did not show any functional connection between striatum and SN during the same behavioral setting. Further behavioral experiments with an implanted stimulator will help to analyze the effects of STN-DBS on motor impairments. The fact that unilateral STN-DBS strongly influences the non-stimulated hemisphere is clinically relevant, in particular for PD patients with strong unilateral predominance of motor impairments. In principle, it could well be that the connection to the ‘healthy’ SN provides a beneficial compensatory mechanism that could depend upon a highly specific stimulation localization as well as highly specific stimulation parameters with respect to amplitude pulse width and frequency. Given the latest generation of brain stimulation devices with directional multi-source stimulation possibilities, it is conceivable that such very specific mechanisms can be targeted. However, profound experimental work has to be carried out to understand this phenomenon before a reasonable transfer into stimulation settings in patients with PD is reasonable and justified.

## MATERIALS AND METHODS

### Experimental design

Thirteen adult male Long Evans rats (*Rattus norvegicus*, Janvier Labs; 328-365 g, 3 months old) were used for DBS. Seven of them received a dopaminergic lesion with 6-OHDA (see below), six were sham-operated. With every animal, one [^18^F]FDOPA- and two [^18^F]FDG-PET scans (DBS ON and OFF) were performed. The following timeline was applied. Day 0: dopaminergic or sham lesion (6-OHDA or vehicle injection) and implantation of the guide cannula. Day 13-24: [^18^F]FDG-PET scans in ON and OFF condition; each animal was measured on two different days, one without electrode (OFF condition) and the other with continuous DBS (ON condition) in a randomized order. Day 26-29: [^18^F]FDOPA-PET scan without electrode (OFF condition).

Differences between 6-OHDA rats and shams with regard to OFF state [^18^F]FDG- and [^18^F]FDOPA-uptake have previously been reported (Kordys et al., 2017). This work is focused on the effects of STN-DBS (ON versus OFF) on [^18^F]FDG-uptake. In addition, 19 healthy rats served as ‘normative population’ for seed analysis based on resting state [^18^F]FDG scans. Rats were housed in pairs in individually ventilated cages (NextGen, Ecoflow, Phantom; Allentown) under controlled ambient conditions (22±1°C and 55±5% relative humidity; data are mean±s.d.) on a reversed 12 h light/dark schedule (lights on 20:30-8:30). They had free access to food and water. Experiments were carried out in accordance with the EU directive 2010/63/EU for animal experiments and the German Animal Welfare Act (TierSchG, 2006) and were approved by regional authorities [Ministry for Environment, Agriculture, Conservation and Consumer Protection of the State North Rhine-Westphalia (LANUV NRW)].

### Surgery

Animals were anesthetized [initial dosage: 5% isoflurane in O_2_/N_2_O (3:7), reduced to 1.5-2.5% isoflurane for maintenance] and received 0.1 ml Carprofen (Rimadyl^®^, Pfizer^®^) subcutaneously as pain relief. Each animal was fixed on a warming pad in a stereotactic system with a motorized stereotactic drill and injection robot (Robot Stereotaxic, Neurostar^®^). For lesion generation, 21 µg 6-OHDA hydrobromide (stabilized with ascorbic acid; Sigma-Aldrich) in 3 µl NaCl was injected unilaterally into the medial forebrain bundle (*n*=7; coordinates: −4.4 mm posterior, 1.2 mm lateral, 7.9 mm ventral to Bregma). In sham animals (*n*=6), 3 µl NaCl was injected into the same intracerebral location. Injection side (left/right) was randomized. Subsequently, a plastic guide cannula (outer diameter 0.71 mm, inner diameter 0.39 mm, length 8 mm; PlasticsOne^®^) was implanted into the injected hemisphere (−3.6 mm posterior, 2.8 mm lateral), targeting the STN for DBS in both 6-OHDA lesioned and sham animals.

### MRI measurements

On the day after surgery, the correct placement of the guide cannula was confirmed using MRI. Imaging was conducted on a 7T Biospec 70/20 USR MRI scanner (Bruker Biospin) equipped with a BGA12S-HP gradient set with a maximum gradient strength of 450 mT/m. Rf-coils were used in a cross-coil configuration of an actively decoupled linear transmit-only resonator with 7 cm inner diameter and a custom-built, anatomically shaped single turn receive-only surface coil of 3 cm diameter. The receive coil was placed on top of the head with the pedestal of the implanted guide cannula extending through the loop of the surface coil. High-resolution anatomical imaging of the site of implantation was carried out using a TurboRARE sequence with five slices (each slice 500 µm), a square matrix of 256×256 pixel in a field of view of 32×32 mm^2^ resulting in an in-plane resolution of 125 µm in each direction. The echo time (TE) was set to 12 ms, RARE factor was 8, resulting in an effective TE of 36 ms. The repetition time (TR) was set to 5700 ms and eight averages were acquired. Acquisition time was 24 min.

### Stimulation

Bipolar 38 mm tungsten electrodes (Microprobes^®^, 1 MΩ, 0.216 mm outside diameter) were used for stimulation. Each animal had its own electrode exactly targeting the STN. The insertable length was restricted by a glue bubble at the electrode shaft, which was individually placed according to the magnetic resonance images. Stimulation sites are shown in Fig. 1.

Immediately before stimulation, the animal was anesthetized with isoflurane (see above) and the cap of the guide cannula was removed. The electrode was inserted with the help of a stereotactic microdrive under stereo-microscopic control. It was important that the delicate tips of the electrode did not touch the rim of the guide cannula, which would cause bending. After the electrode was slowly advanced to its final depth, it was fixed to the pedestal. The whole insertion process took ∼5 min. The animal was then transferred to a chamber of 30×24×21 cm, which was equipped with a video camera (Logitech webcam) and infrared LEDs. Before the rat awoke, the electrode was connected to a Master-8 stimulus isolator (A.M.P.I., Jerusalem, Israel) via a swivel, which allowed movement and turning of the rat inside the chamber. Stimulation was switched on, and monophasic rectangular 60 µs pulses were delivered at 130 Hz (Volkmann et al., 2006). The amplitude was initially set to 30 µA and then slowly increased to 50 µA in 5 µA steps. When side effects (teeth grinding, gagging movements) appeared, the amplitude was lowered to 5 µA below side effect level. All stimulation currents were between 30 and 50 µA.

### PET measurements

#### [^18^F]FDG with stimulation

[^18^F]FDG-PET experiments were performed 13-24 days after 6-OHDA or sham injection. They were combined with the first stimulation the rat ever received. After 15 min of stimulation in the chamber, 72.4±4.7 MBq of [^18^F]FDG was injected intraperitoneally without interrupting the connection to the stimulator. After 40 min, the animal was disconnected and anesthetized. The electrode was removed, the guide cannula recapped, and the rat was fixed on an animal holder (Medres^®^) with a respiratory mask. Body temperature was maintained at 37°C using a feedback-controlled warming system (Medres^®^). A 30 min emission scan was started 60 min after [^18^F]FDG injection using a Focus 220 micro PET scanner (CTI-Siemens). This was followed by a 10 min transmission scan using a ^57^Co point source for attenuation correction. Summed images (60-90 min post-injection) were reconstructed using the iterative OSEM3D/MAP procedure (Qi et al., 1998) resulting in voxel sizes of 0.38×0.38×0.82 mm. Further analysis was carried out using the VINCI Software (version 4.92, Max Planck Institute for Metabolism Research; available at vinci.sf.mpg.de). Images were co-registered manually to the Swanson rat brain atlas (Swanson, 2004). When lesions were in the right hemisphere, images were flipped so that the intervention was always displayed on the left. Intensity was divided by the cerebral global mean (standardized uptake value ratio, SUVR_wb_).

#### [^18^F]FDG without stimulation

These measurements took place in the same time period, but on a different day as the stimulation experiments. No electrode was inserted. After 15 min in the chamber, 73.0±3.7 MBq of [^18^F]FDG was injected intraperitoneally in the awake animal. The rat remained in the chamber for 45 min and was subsequently anesthetized and measured as described above.

#### [^18^F]FDOPA

[^18^F]FDOPA-PET measurements and results from the animals used in this study are described in detail elsewhere (Kordys et al., 2017). Striatal [^18^F]FDOPA uptake normalized to cerebellum was used to calculate dopamine depletion severity as 1-(SUVR_cer_ipsi/SUVR_cer_contra).

### Analysis of DBS effects

From each animal (6-OHDA, *n*=7; sham, *n*=6) we received two [^18^F]FDG images: with (ON) and without (OFF) stimulation. ON and OFF images were compared voxel-wise, separately for 6-OHDA and sham animals, using a paired *t*-test. This was followed by a threshold-free cluster enhancement (TFCE) procedure with subsequent permutation testing, resulting in a statistical map corrected for multiple testing, thresholded at *P*<0.05 (Smith and Nichols, 2009). Because TFCE values are arbitrary, color bars of TFCE maps were labeled with the original *t*-values, marked *t*_TFCE_ (Fig. 2A). To assess whether DBS affected the brains of 6-OHDA and sham animals differently, the subtractive images (ON minus OFF) were compared between groups. An unpaired *t*-test followed by TFCE and permutation testing was used (Fig. 2B). To examine whether DBS effects were related to lesion severity, the subtractive images were correlated with dopamine depletion severity values obtained from [^18^F]FDOPA images across all animals. The Pearson correlation test followed by TFCE and permutation testing was used. The color bar of the statistical map was labeled with the original correlation coefficients, marked as R_TFCE_ (Fig. 2D).

### Functional connectivity analysis

To study whether dopamine depletion and DBS altered striatal functional connections, seed-based metabolic connectivity analyses (Rohleder et al., 2016; Yakushev et al., 2017) were performed with 6-OHDA animals, separately for ON and OFF conditions. To compare it with the healthy state, we used 19 healthy control rats (injected dose≈75 MBq [^18^F]FDG, awake uptake under the same conditions). [^18^F]FDG images were smoothed with a Gauss kernel of 1.5 mm full width at half maximum (FWHM), and two seed regions (four voxels each) were chosen: one in the middle of the intact (contralesional) striatum, the other in the ipsilesional striatum at the spot where DBS led to increased FDG uptake. In control rats, seeds were placed accordingly in the middle of the right striatum and in the left ventrolateral striatum. The images were correlated voxel-wise with the mean SUV_wb_ of the seed region, using a Pearson correlation test. The resulting R-maps were TFCE-corrected as described above.

### Histology and immunohistochemistry

Correct placement of the electrode with the help of magnetic resonance images as well as effectiveness of the 6-OHDA dopaminergic lesion was confirmed using histology and immunohistochemistry in four animals. Rats were transcardially perfused with 4% paraformaldehyde in PBS (pH 7.4). The brain was removed and cut into 35 µm transverse sections using a cryostat (Leica CM1950). Sections were alternately placed on microscope slides designated for histology or tyrosine hydroxylase (TH) immunohistochemistry. For TH immunohistochemistry, sections were incubated with 0.5% H_2_O_2_ in distilled water for 1 h, followed by incubation with normal horse serum (three drops in 10 ml PBS; Vectastain Elite ABC kit)+0.5% Triton X-100 for 1 h. The primary antibody, monoclonal mouse-anti-TH (Sigma-Aldrich, catalogue no. T1299, clone TH-2) was applied in a concentration of 1:5000 in PBS together with 0.1% normal horse serum overnight. Biotinylated anti-mouse IgG was used as secondary antibody (two drops in 10 ml PBS; Vectastain Elite ABC kit, Vector Laboratories, catalogue no. PK-6102) and incubated for 1 h. Meanwhile, the avidin-biotin-horseradish peroxidase (HRP) complex was prepared by adding two drops of avidin (solution A; Vectastain Elite ABC kit) and two drops of biotinylated HRP (solution B; Vectastain Elite ABC kit) to 10 ml PBS. After washing off the secondary antibody, sections were incubated in the A-B solution for 30 min. Finally, binding sites of the primary antibody were visualized using diaminobenzidine (DAB) with CoCl_2_ intensification (Sigmafast DAB with metal enhancer; Sigma-Aldrich). One DAB tablet was dissolved in 10 ml distilled water and a urea hydrogen peroxide tablet (included in the Sigmafast package) was added. Sections were incubated for ∼10 min, washed in PBS, dehydrated in ethanol and Roti-Histol (Carl Roth), and coverslipped in Entellan (Sigma-Aldrich). For histology, sections were stained with Hematoxylin and Eosin, dehydrated and coverslipped.
